# Interface properties of nanostructured carbon-coated biological implants: an overview

**DOI:** 10.3762/bjnano.15.85

**Published:** 2024-08-16

**Authors:** Mattia Bartoli, Francesca Cardano, Erik Piatti, Stefania Lettieri, Andrea Fin, Alberto Tagliaferro

**Affiliations:** 1 Center for Sustainable Future Technologies (CSFT), Istituto Italiano di Tecnologia (IIT), Via Livorno, 60, 10144, Torino, Italy; 2 Consorzio Interuniversitario Nazionale per la Scienza e Tecnologia dei Materiali (INSTM), Via G. Giusti 9, 50121, Firenze, Italy; 3 Department of Chemistry, University of Turin, Via P. Giuria 7, 10125 Torino, Italy; 4 Department of Applied Science and Technology, Politecnico di Torino, Corso Duca Degli Abruzzi, 24, 10129, Torino, Italy

**Keywords:** biocompatibility, carbon nanotubes, coatings, graphene, nanodiamonds, surfaces

## Abstract

The interfaces between medical implants and living tissues are of great complexity because of the simultaneous occurrence of a wide variety of phenomena. The engineering of implant surfaces represents a crucial challenge in material science, but the further improvement of implant properties remains a critical task. It can be achieved through several processes. Among them, the production of specialized coatings based on carbon-based materials stands very promising. The use of carbon coatings allows one to simultaneously fine-tune tribological, mechanical, and chemical properties. Here, we review applications of nanostructured carbon coatings (nanodiamonds, carbon nanotubes, and graphene-related materials) for the improvement of the overall properties of medical implants. We are focusing on biological interactions, improved corrosion resistance, and overall mechanical properties, trying to provide a complete overview within the field.

## Introduction

For centuries, the simple manipulation of natural resources has represented the only available strategy for the realization of artifacts, buildings, and innovations, until the principles laying behind the structure of materials were discovered. The discovery of the atom-based nature of matter has revolutionized the approach to natural science, leading to the development of nanoscience. Noble laureate Richard Feynman first proposed the concept of nanomaterials in his well-known lecture entitled “*There’s Plenty of Room at the Bottom”*, in which he discussed the possibility of the manipulation of individual atoms and molecules [1]. Traditionally, this first lecture was recognized as the birth of nanotechnology, although the term was first used only later by Norio Taniguchi in 1974 to describe the study of materials at the nanoscale [2]. Afterwards, nanosized and nanostructured carbon species have attracted great interest thanks to their intrinsic properties and easy functionalization [3]. The utilization of nanocarbon species has been widely deployed in advanced medical applications [4] as active species or as drug delivery platforms using tailored carbon nanotubes (CNTs) [5–6], fullerenes [7–8], carbon dots (CDs) [9–10], and graphene-related materials (i.e., graphene oxide (GO) [11], reduced graphene oxide (rGO) [12], and nanographite (nG) [13]). Furthermore, the production of nanocarbon-reinforced materials is paving the way for a new era of tissue engineering thanks to their application as high-performance biocompatible scaffolds [14–15] and implantable devices [16–17]. The key features of these materials should be compatible with the complexity of biological environments represented by implant–tissue interfaces [18] through the tuning of different parameters (i.e., surface roughness and potential as well as hydrophobicity).

Cells and biomolecules can selectively adhere to or be repelled from artificial implanted surfaces, triggering several metabolic pathways of high importance [19]. Particularly, cellular adhesion and a controlled immunological response are key features of any artificial device for being effectively implanted [20]. Additionally, responsive surfaces represent the last frontier in nanomedicine, and they require the exchange of signals and information at the molecular level with the biological environment [21]. Nanostructured and nanosized materials represent a valid solution to offer all the abovementioned features thanks to their highly controllable properties. Nevertheless, the preparation of nanocarbon-containing materials is still complex because of the efforts required for achieving a homogenous dispersion in an inorganic–organic matrix [22–24].

**Figure 1:**
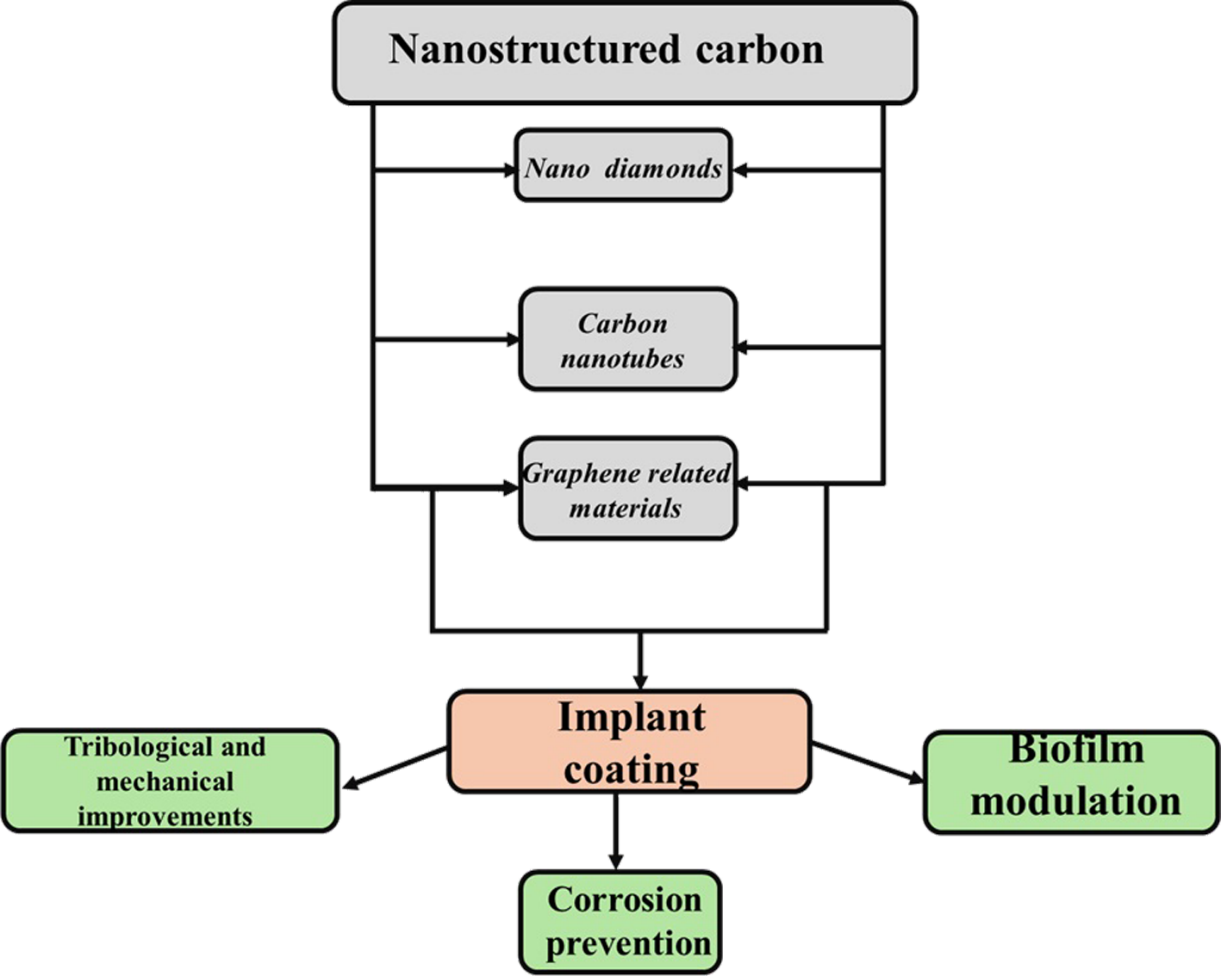
Relation between nanostructured carbon materials and the tuned properties in biological implants.

In this short review, we are discussing nanostructured and nanosized carbon-based materials used to improve the durability and physicochemical properties of biological implants as summarized in Figure 1.

## Review

### Nanostructured carbon-containing materials at biological interfaces

Interfaces between artificial and biological environments play a critical role for the design and long-term performance of any artificial implant [25–26]. The interface between an implant and the biological environment is a dynamic and complex area, where several biological, physical, and chemical interactions can take place simultaneously, including immunological response [27], mechanical mismatch with the tissue [28], degradation [29], responses to stimuli [30], and proliferation of bacteria [31]. In the next section, we investigate the effect of carbon-based nanostructured interfaces on the mitigation of adverse effects occurring to biological implants, considering advantages and disadvantages as summarized in Table 1.

**Table 1:** Summary of advantages and disadvantages of the utilization of nanostructured carbon interfaces in biological implants.

Nanostructured material	Advantages	Disadvantages

nanodiamonds	* increased wear stress resistance	–
carbon nanotubes	* increased wear stress resistance	–
graphene and related materials	* increased wear stress resistance	–

### Nanostructured and nanosized carbon materials: an overview

**Figure 2:**
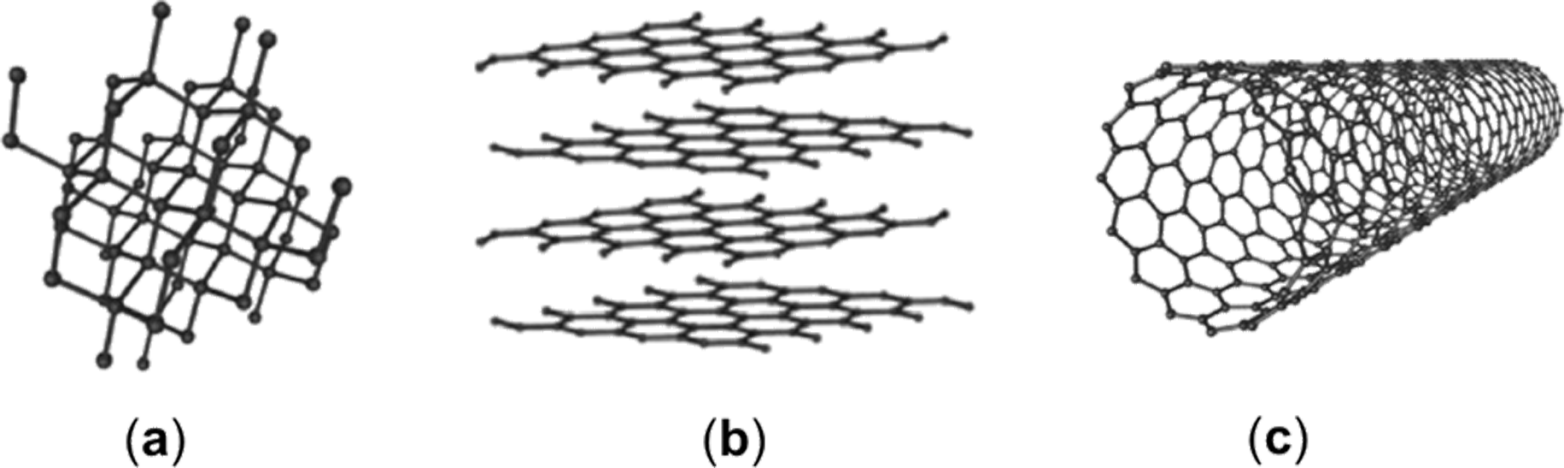
Summary of carbon allotropes: (a) NDs, (b) graphene-related materials, and (c) CNTs. Figure 2 was adapted from Wikimedia Commons https://commons.wikimedia.org/wiki/File:Eight_Allotropes_of_Carbon.png (created by Michael Ströck (mstroeck), distributed under the terms of the CC BY-SA 3.0 Unported License, http://creativecommons.org/licenses/by-sa/3.0).

The family of nanostructured carbon materials has several members with peculiar properties, namely, (i) graphene-related materials, (ii) CNTs, and (iii) nanodiamonds (NDs) as shown in Figure 2.

All nanosized carbon materials show remarkable properties regarding both thermal and electronic conduction, but they should be treated carefully to ensure reproducible production protocols. The next section briefly overviews all nanosized carbon materials since a deep understanding of each material is of capital importance for a comprehensive understanding of their applications at biological interfaces.

#### Graphene and graphene-related materials

In 2008, Lee and co-workers [32] stated that neat graphene was the strongest material ever tested with a tensile strength of 131 GPa and a Young’s modulus close to 1 TPa. The reason for these properties of graphene is the stability of the π-bond network around the hexagonal structures of carbon rings, which prevents planar deformations [33]. The same phenomenon explains the high thermal conductivity of up to 3000 W·m^−1^·K^−1^ [34–35] and the outstanding electrical properties [36–38].

Compared to conventional 3D materials, the understanding of electronic transport and carrier dynamics in graphene is significantly complicated by the extreme anisotropy intrinsic to its crystal structure and its large compositional and structural variability [39]. Beyond the obvious consequences arising from the chemical composition, some of the main aspects affecting electronic transport in graphene are structural polymorphism [40] (arrangement, number, and order of layers), the electronic coupling between different layers, and the matrix in which graphene is embedded [41]. Indeed, the matrix plays a pivotal role owing to the ultimate surface-to-volume ratio and the poor electrostatic screening displayed by graphene-based composites [39]. These aspects are especially relevant in determining the in-plane electronic transport within each layer of graphene (intra-layer transport). Conversely, the electronic coupling between different layers dominates the out-of-plane electronic transport from one layer to another (inter-layer transport) and is the source of the large anisotropy typically displayed between in-plane and out-of-plane carrier mobilities [42]. Carrier injection is usually determined by energy band alignment and interface transparency, and it is limited by the number of available conduction pathways and the existence of a tunnel barrier between graphene flakes [43]. Accordingly, the transparency of the interface between different flakes determines whether the inter-flake transport is diffusive or hopping-type regardless of the intrinsic mechanisms responsible for intra-flake transport in graphene-containing materials [44].

Furthermore, the manipulation of pristine graphene is a hard task, and several derivatives (i.e., GO and rGO) have been developed to partially preserve the properties of pristine graphene while enabling better handling.

GO is an oxidized graphene derivative rich in oxygen functionalities (hydroxy, epoxy, carbonyl, and carboxylic groups) arranged according to the Lerf–Klinowski model [45]. GO is generally produced through chemical oxidation and exfoliation of graphite flakes with different protocols to tune the oxygen content [46–47]. The defective structure of GO deeply affects its electronic properties, which are considerably inferior compared with neat graphene. However, GO can be suspended in several solvents and easily functionalized to act as a chemical platform [48]. rGO stands as a compromise between the easier handling of GO and the properties of neat graphene. rGO is produced through direct reduction of GO using physical or chemical routes [49]. Thus, the carbon-to-oxygen ratio can be increased to values of around 8:1 to 246:1, significantly higher than those of GO [50]. The electrical properties show a remarkable improvement compared with GO, even if they still remain far below those of graphene. Last, GO and rGO show good interactions with polymeric matrices thanks to specific surface functionalizations [51].

#### Carbon nanotubes

CNTs are an allotropic state of carbon discovered in the middle of the 20th century [52–55], which became famous in 1991 [56]. CNTs can be described as single or multiple cylindrical graphite sheets rolled up in a tubular structure forming single-walled CNTs (SWCNTs) or multiwalled CNTs (MWCNTs). SWCNTs are characterized by diameters from 0.3 nm [57] to 1 nm [58], while the diameters of MWCNTs can reach 100 nm [59–60] with a very high aspect ratio. The length of CNTs varies from a few nanometers [61] to several centimeters [62], and it is strictly related to the synthesis method. Additionally, CNTs can end with fullerene-type caps that are highly reactive because of the high distortion [63].

As summarized in Table 2, individual SWCNTs or MWCNTs show incredibly good mechanical and conduction properties. Nevertheless, individual CNTs are rare, and most applications are based on CNT bundles, which are difficult to homogenously disperse in polymeric matrices, and whose properties are not comparable with those of individual CNTs [71].

**Table 2:** Summary of properties of single SWCNTs and MWCNTs.

	Young’s modulus (GPa)	Tensile strength (GPa)	Resistivity (Ω·m)	Thermal conductivity (W·m^−1^·K^−1^)

SWCNTs	900–1700 [64]	75 [65]	10^−6^ [66]	1750–5800 [67]
MWCNTs	690–1800 [68]	150 [65]	10^−5^ [69]	3000 [70]

#### Nanodiamonds

NDs are a carbon allotrope composed by sp^3^-hybridized carbon atoms arranged in a tetrahedral crystalline lattice structure [72]. The structure is accountable for the high thermal conductivity due to efficient heat conduction through phonon vibrations, which can reach 550 W·m^−1^·K^−1^ after sintering at high pressure [73]. Nevertheless, surface defects and the granular shape of the NDs represent boundaries for phonon transport reducing the thermal energy propagation [74]. Furthermore, the thermal conductivity of NDs increases with the increment of temperature because the higher number of phonons increases the efficiency of thermal transport [75]. NDs show also exceptional mechanical strength and low chemical reactivity, making them sound candidates for thin film coatings [76]. These properties are counterbalanced by a low electrical conductivity due to quantum confinement as reported by Bolker and co-workers [77]. Authors reported that the bandgap of NDs is strongly correlated to the NDs’ size, and it increases with decreasing crystallite size. However, the ND properties can be altered by heteroatomic doping and through the introduction of surface defects, including passivation and vacancies [78].

### Deposition methods for the synthesis of carbon coatings

The addition of nanostructured and nanosized carbon species into materials for biological applications can be attained by several techniques such as chemical vapor deposition (CVD), physical vapor deposition (PVD), and in situ formation through laser treatments. CVD offers several advantageous features such as a high degree of control over the deposition process. CVD involves the deposition of a thin film of material onto a substrate through homogeneous or heterogeneous reactions [79]. Homogeneous reactions are those involving the decomposition of precursor in the gas phase forming products that condense on a target. In contrast, heterogeneous reactions are those involving the decomposition of the precursors on the solid surface of a catalyst that also acts as a support. As reported by Porro et al. [80], the variation of few parameters (i.e., precursor flux and process temperature) can be sufficient to obtain nanographite or CNTs via CVD. Furthermore, Musso et al. [81] proved that, under appropriate conditions, CNTs and carbon microfibers can be grown from different carbon precursors (i.e., camphor and cyclohexanol) on various substrates, ranging from uncoated silicon to simple glass, to yield carpets of vertically aligned CNTs. Nevertheless, a purification stage for removing the catalyst is mandatory for avoiding side effects in biological environments [82–83]. The choice of the catalyst is strictly related to the desired carbon nanomaterials. Metal catalysts with high carbon solubility primarily involve carbon segregation and precipitation throughout the metal bulk [84], while metal catalysts with low carbon solubility act from the metal surface inward [63].

PVD routes are numerous, and they are classified according to the power sources used for the process (i.e., plasma-, direct current-, radiofrequency-, and ion beam-assisted coatings) [85]. All PVD processes are based on a vacuum chamber containing the material to be deposited, known as target, and the chosen substrate onto which the deposition occurs. During electron beam evaporation, an electron beam is used to vaporize the target material, while during sputtering, a high-energy ion beam is used to bombard the target. In both cases, atoms are ejected from the target and subsequently condense onto the substrate. The thickness and some morphological properties of the deposited nanostructured film can be controlled by adjusting deposition time, substrate temperature, and deposition rate.

### The response of biological surfaces to non-biological materials

**Figure 3:**
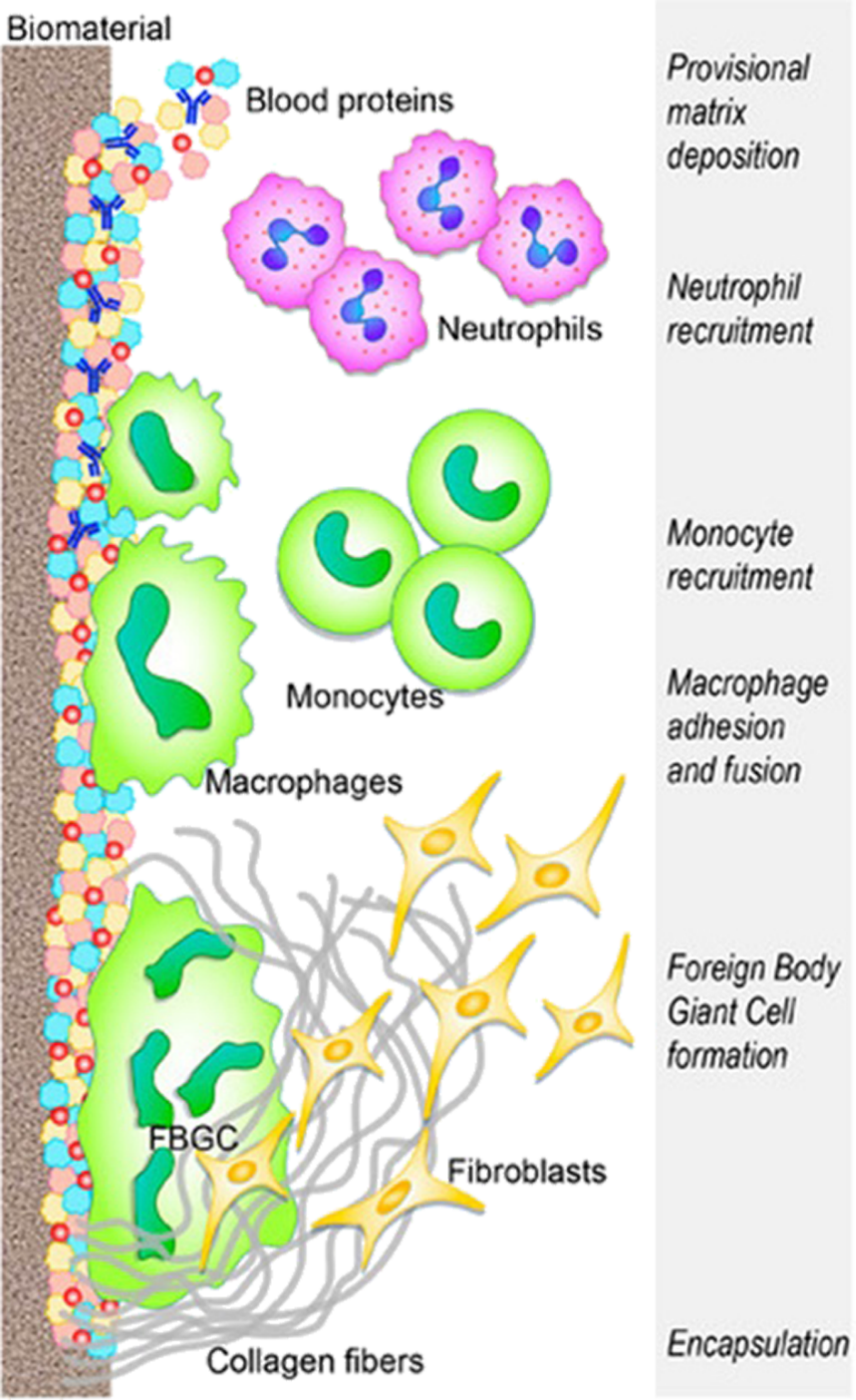
Summary of the immune system response to implanted biomaterials. Figure 3 was reproduced from [86] (© 2019 E. Mariani et al., published by MDPI, distributed under the terms of the Creative Commons Attribution 4.0 International License, https://creativecommons.org/licenses/by/4.0).

The first challenge in developing biomedical implants is related to biocompatibility because the implant interfaces are the first line of contact between a foreign body and living organisms. The bulk materials used in implants are carefully chosen to minimize adverse reactions, but the immune system may still recognize them as external entities and trigger inflammatory responses due to mere surface interactions, as summarized in Figure 3.

The interaction between implants and the immune system is highly tissue-specific, with different responses observed depending on the implantation sites. Usually, the insertion of an implant is followed by the adsorption of plasma components onto the surface, forming a matrix composed by platelets and coagulation cascade components. This process triggers the inflammatory response of neutrophils, which attempt to degrade the implant through phagocytosis and the release of reactive oxygen species. Macrophages play a key role changing from pro-inflammatory to anti-inflammatory phenotypes. The final step in the immune system response involves the formation of foreign-body giant cells on the implant surface. Increased cytokine levels trigger the release of pro-fibrogenic factors and recruiting fibroblasts. Fibroblasts induce the accumulation of collagen leading to the foreign-body reaction. This phenomenon involves the formation of a fibrous capsule around the implant, which compromises its functionality (such as the flexibility of cardiovascular stents) and, thus, limits the integration of soft tissue implants.

#### The effect of nanostructured carbon on surfaces on the biological response

The modulation of the implant–tissue interface is a complex field of work, which involves both chemical and biological issues. The addition of nanostructured carbonaceous layers represents a solid choice because of the intrinsic properties of carbon materials and the possibility to further tailor them with simple chemical modifications [87–88].

Thomas et al. [89] investigated ND coatings as active surface for tuning the macrophage response to estimate the long-term inflammatory effects of wear debris. The authors investigated the effect of the ND particle sizes on macrophage proliferation, platelets aggregations, and inflammatory cytokine release. NDs significantly reduced the concentration of platelet-derived growth factor compared to serum. Also, there was a complex dependence of macrophage responses on local concentration and size of the NDs, suggesting that the ND coating prevented the removal of wear debris from coated implants. The complex relation between NDs and macrophage activity can be further tuned by functionalization with short molecules as reported by Pentecost and co-workers [90], who used small amines to tune the inflammatory response. The mechanisms involved in inflammation related to NDs are not yet clear, but authors suggest that the process is started by serum protein deposition triggering the inflammatory cascade. Moreover, polymeric films containing NDs were optimum substrates for osteoblast proliferation as reported by Mansoorianfar and co-workers [91]. Booth et al. [92] dealt with foreign-body giant cell formation, disproving that significant changes in wettability and surface energy affect the in vivo effects of NDs on titanium surfaces. The authors surprisingly reported a negligible effect of ND coating on fibrous formation, suggesting that, in the specific case, NDs act only as protective layer rather than influencing the immunological response.

GO-modified surfaces exhibited better performance in tuning the immunological response, as reported by El-Kamel et al. [93], who coated AZ91E Mg alloy staples used for gastrectomy surgery. The GO coating enabled, at the same time, improved corrosion resistance, high cell proliferation, and very low inflammatory response. Similarly, Fernández-Hernán et al. [94] used graphene nanoplatelets to coat AZ31 magnesium, evaluating cytocompatibility, osteoblasts adhesion, and proliferation. The authors reported a significant improvement of cytocompatibility with the creation of a preosteoblastic monolayer on the coated surface after one week of cell culture. Chen and co-workers [95] investigated the effect of GO coating as antifibrotic on metal implants. They controlled the roughness, inducing macrophage polarization to the pro-inflammatory state without producing a great excess of pro-inflammatory factors. Furthermore, GO-coated implants showed a reduction of the expression of the fibrosis-related protein α-SMA and collagen deposition in the presence of both fibroblasts and macrophages. The reduction of fibrotic formations on the implants is of capital relevance for preventing thrombosis [96]. Hassan et al. [97] investigated graphene coatings on a stainless steel implant to minimize the negative effect of metals contained into the alloy (i.e., Cr, Mo, and Ni). The authors used PVD for producing the coating, evaluating both hemolysis and blood coagulation to assess the antithrombotic properties of the graphene coating. The coated implant showed a higher hydrophobicity with less adhered platelets and a 70% reduction of hemolysis. As mentioned, the hydrophobicity and low reactivity of carbon coatings are the key features for the lowered immunological response.

Contrary to NDs and graphene-related materials, CNT layers generally induce a strong immunological response because of their higher reactivity [98], which needs to be tuned through an appropriate functionalization tailored to the tissues where the implant will be placed [99]. Nevertheless, CNTs are able to regulate the cell proliferation better than other nanocarbon species. Patel et al. [100] coated polymer nanofibers with a 25 nm thick layer of MWCNTs modulating in vivo angiogenesis and bone regeneration. Furthermore, the authors were able to fine-tune the topology of the CNT coating, reducing inflammatory events by down-regulated pro-inflammatory cytokines and macrophages. The coated polymeric nanofibers showed the ability to up-regulate the formation of new blood vessels and osteogenic pathways, proving the key role of the CNT coating topology in the compatibility with living tissues.

### The formation of biofilms and the microbial proliferation on implants surfaces

**Figure 4:**
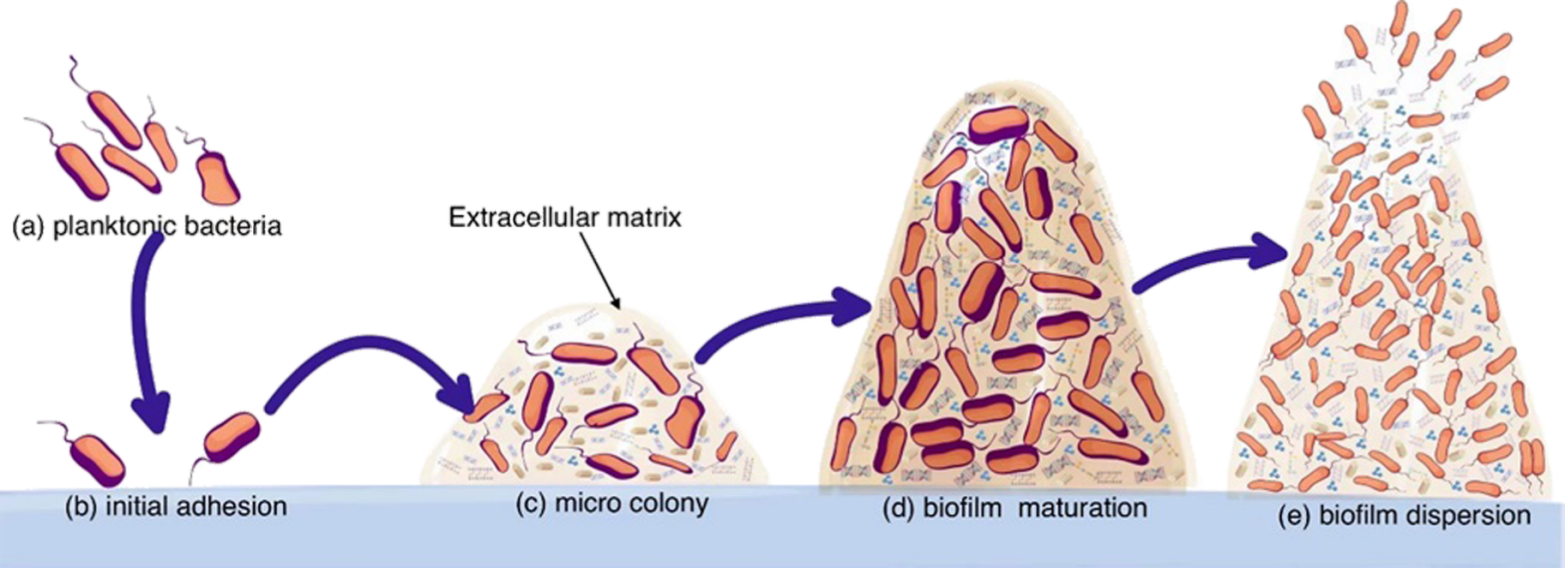
Biofilm formation on an implanted biomaterial due to the presence of planktonic bacteria cells. Figure 4 was reproduced and adapted from [104] (© 2022 R. Ma et al., published by Frontiers, distributed under the terms of the Creative Commons Attribution 4.0 International License, https://creativecommons.org/licenses/by/4.0).

The interface between implants and tissues is a key vulnerable point for infection spreading because of the formation of bacteria biofilms [101]. Generally, gram-positive bacteria are the most common culprits for implant infections, while aerobic gram-negative ones seldomly are [102–103]. Furthermore, the biofilm formation is strongly correlated with the implantation site, and the spreading time of infection mainly depends on the virulence of bacteria according to the mechanism shown in Figure 4.

The biofilm formation consists of four stages, namely, (i) adhesion, (ii) aggregation, (iii) maturation, and (iv) dispersion. Adhesion is the first step and is ruled by the polarity of the surface as reported by Gittens and co-workers [105]. The aggregation and maturation steps involve the formation and enlargement of bacteria colonies enclosed in the extracellular biopolymeric matrix [106]. Dispersion occurs upon reaching the biofilm’s critical mass, allowing for the partial detachment and spread of infection through the bloodstream [31].

#### Carbon materials coatings for mitigation of biofilm formation

Biofilm formation represents a major concern because of the severe actions needed to restore or remove the damaged implant [107]. Prevention of biofilm proliferation can be achieved through the development of coatings changing the hydrophobicity of the implant surface using several routes [108–109], including the incorporation of nanostructured carbon species [101]. Graphene and graphene-related materials have been widely used as antimicrobial coatings for several kinds of implants to tune the surface hydrophobicity and to prevent bacteria adhesion [110–111]. Romo-Rico et al. [112] used PVD to coat a medical-grade cobalt–chromium alloy with high-grade graphene. The authors reported an appreciable antibacterial activity against *S. aureus* and *P. aeruginosa*; also, adhesion was prevented. This study proved that the balance between surface polarity and bacteria targeting is crucial for engineering coating solutions. Similarly, Al-Saadi et al. [113] used CVD for coating a nickel–copper alloy with multilayered graphene, showing the effectiveness of carbon coatings in replacing the native protective oxide layer of the alloy and in reducing the adhesion of sulfate-reducing bacteria. Furthermore, graphene coatings can also exhibit antibacterial activity through electron transfer phenomena as reported by Yang et al. [114] for graphene coatings on titania. The authors reported that the increased electrical conductivity was due to the unpaired electrons at the Schottky-like interface between graphene and titanium. The enhancement of electron transfer rate promoted a relevant bactericidal action. Furthermore, the authors proved the relationship between activity and electron transfer rate by adding an insulating layer of zirconia and observing no bactericidal effects. The authors also proved the cytocompatibility of the bactericidal coatings. GO showed similar results on titanium surfaces as reported by Yang et al. [115], reporting antibacterial activity of over 99% against both *E. coli* or *S. aureus* when a small doping with copper was applied. The synergistic effects of metal cations in GO coatings were extensively investigated [116–117]. Also, polymer blends were included instead of metal species [118–119].

ND coatings are also of particular interest for the prevention of biofilm formation [120] because of the reduced bacteria adhesion, which interferes with microbial film formation [121]. As reported by Rifai et al. [122], ND coatings can be easily applied to titanium surfaces, creating a hydrophilic surface to reduce the adhesion of *S. aureus*. Despite the lowered adhesivity, pristine NDs do not show any significant antibacterial activity. In contrast, functionalized oxidized ND layers were able to inhibit the growth of *E. coli* comparable to the effect of ampicillin [123]. Similarly, mannose ND coatings interfered with the proliferation of uropathogenic bacteria, representing a solid choice to prevent catheterization [124–125] and targeting the FimH protein complex involved in bladder infection.

CNTs also prevent the formation of biofilm as reported by Sivaraj et al. [126], who obtained zones of inhibition of up to 12 mm. Morco et al. [127] suggested that the biofilm inhibition by CNTs is mainly due to the increase of surface hydrophobicity and nanostructuring [128–129]. Kang et al. [130] suggested that the main mechanism of action is cell disruption due to the mechanical effect of CNTs themselves via surface polarity changes. Rodrigues et al. [131] dwelled more deeply into CNT coatings and found a correlation between the exopolymer substances secreted and the effectiveness of CNT action.

### Carbon material coatings for improved mechanical, tribological, and electrical properties

Performance and longevity of implants are closely related to their mechanical properties. A mismatch with tissues can potentially lead to stress shielding, wherein the implant bears an excessive load, consequently, causing bone resorption [132]. Coating with nanostructured carbon is a strategy to both reduce wear and improve load across the implant region. As mentioned by Zhang et al. [133], a nano- or micrometric thick layer of CNTs induced the ability of self-repairing of the damaged surfaces by filling the cracks, thus, reducing wear loss. Chen et al. [134] improved the mechanical properties of a titanium alloy though deposition of graphene flakes. The authors investigated the system through indentation showing improvements in both toughness and yield strength. The improvements are due to the graphene coating, which allowed for a better load transfer, inter-layer sliding, and crack deflection. Similarly, Askarnia et al. [135] used electrophoretic deposition for coating a magnesium alloy with GO. The authors reported an increase of both hardness and Young’s modulus of 100% and 156%, reaching 60 MPa and 0.52 GPa, respectively, after coating. CNTs are generally used to reinforce the bulk of composite-based implants [136] or added to polymeric films [135]. Interestingly, they can be mixed with hydroxyapatite in order to magnify the compatibility with bone tissues [137] to reduce wear. Such layers have been widely studied as coating agents onto several metal surfaces directly in contact with bone, including steel [138], titanium [139], and magnesium [140]. As reported by Deenoi et al. [141], CNT coatings on titanium nitride at the interface with ultrahigh-density poly(ethylene) reduced the friction coefficient more than any other tested nanostructured carbon coating.

Nevertheless, surface wear remains an issue that needs to be solved in several key implants such as cardiovascular devices and joint replacements. NDs can play a crucial role because of the superior friction reduction achievable using thin ND films as reported by Blum and co-workers [142]. The authors sintered a 75 µm thick layer of NDs onto an aluminum alloy using a focused laser beam and reached a friction coefficient smaller than 0.2. Similar results can be obtained by using PVD, CVD, and sol–gel deposition of NDs on ceramics, together with excellent adhesion of the protective layer [143–144].

Chernysheva et al. [145–146] investigated the production of protective ND layers onto a xenogenic heart valve, evaluating the role of the ND surface potential. A negative surface potential influenced the mechanical characteristics, suggesting a better interaction with the surrounding tissues. Jozwik et al. [147] also reported the long durability of ND coatings in heart implants without any appreciable decrement of performance. Furthermore, ND layers can be easily integrated with other carbon-rich parts of a heart valve implant, suppressing thrombin generation from platelets as reported by Zeng and co-workers [148].

ND coatings are also able to boost the integration of implants with tissue as reported by Zalieckas and co-workers [149]. The authors coated a titanium alloy with NDs by CVD at 400 °C and observed good proliferation of osteogenic cells on a bone implant, even better than on a commonly used surface. They suggested that the osteoblast proliferation was mainly due to the surface morphology and the good match between cells and surface potentials.

### Carbon-based material coatings for the prevention of corrosion

Corrosion of metallic implants is still a major concern regarding loss of integrity, thrombosis, and inflammatory processes [150]. To date, the development of highly corrosion-resistant alloys has not been satisfactorily achieved, although coating is a solid choice to prevent massive implant degradation. Carbon nanomaterial coatings can prevent adverse chemical reactions triggered by both the adsorption of proteins and the metabolism of cells [151–153]. Hassan et al. [97] extensively investigated the effect of graphene and graphitic coatings as both anticorrosion and antithrombotic elements. The authors used PVD for the deposition of a micrometer-thick coating on stainless steel, controlling morphology, roughness, and mechanical parameters. The coated surfaces showed a reduction of hemolysis of 40% and a corrosion resistance increment of 96% compared with the untreated surface. The authors suggested that the improved performance was due to the changes adsorption rate of protein and plasma compounds. Mallik et al. [154] used electrophoretically deposited graphene for coating titanium, achieving a strong reduction in corrosion with a coating thickness of 12 μm. The same technique was used by Chen et al. [155] for the deposition of GO onto magnesium alloys. The authors investigated the corrosion in 0.9 wt % NaCl solution, showing beneficial effects of GO mainly due to both being a physical barrier and having a low reactivity. Similar results can be achieved by replacing GO with a mixture of nanostructured calcium carbonate and rGO [156] or fluorohydroxyapatite and GO [157]. Guo et al. [158] also proved the ability of GO coatings to prevent the release of Ni(II) ions from a nickel–titanium alloy during corrosion. The authors suggested that this could be due to both improved corrosion resistance and entrapping of metal ions in the porous structure of GO. Kabir et al. [159] used graphene nanoplates for the coating of zinc implants, achieving a corrosion rate of 0.09 mm/y. Similar results were shown for tantalum [160] and a titanium–aluminium alloy [161].

ND coatings can exhibit the same behavior as graphene-related materials because of their low chemical reactivity. Nezamdoust et al. [162] coated a magnesium alloy with a 20 nm layer of NDs and measured the corrosion rate in Harrison solution by means of electrochemical impedance spectroscopy. The authors observed a drastically reduction of passivation in the coated samples compared with the original specimens.

Similar results can be achieved by using CNT-containing polymeric layers [163] or CNT-based hydroxyapatite coatings [164–165]. Remarkably, neat CNTs coatings are not the best option for anticorrosion layers because of the higher reactivity compared with NDs and graphene-related materials. Nevertheless, the reactivity of CNTs in biological environments is still a topic of great discussion. As mentioned by Fadeel et al. [166], CNTs encompass a wide range of different species with peculiar chemical reactivity and resistance to oxidative stress.

## Summary and Future Perspectives

The results herein discuss the complex scenario of the interaction between implants and living tissues, which is still far from being fully understood. The engineering of implant surfaces with nanosized and nanostructured carbon materials clearly represents a disruptive advancement in the field, leading to prolonged implant life, increased biocompatibility, and reduction of adverse inflammatory reactions.

The big family of low-dimensional carbon materials is a great reservoir for tuning the properties of implants and matching them with those of the tissues. The harsh biological environments, rich of highly reactive and complex species, are a significant challenge for materials science. However, coatings from CNTs, NDs, and graphene-related materials allow one to create smart multipurpose surfaces able to face these issues.

Nanostructured carbon coatings can be groundbreaking in the production of stimuli-responsive implants, such as prosthetic implants. Even if a coherently modulation of nerve signals is still far from being reached, materials such as graphene and CNTs can play a pivotal role in this ambitious long-term goal. Furthermore, the tailoring of carbon surfaces represents a valuable tool in moving from simple implants to medical platforms that are able to monitor and repair themselves, as well as to treat the surrounding tissues.

We firmly believe that the production of specialized carbon surfaces represents a new frontier in the field of durable high-performance implants.

## Data Availability

The data that supports the findings of this study is available from the corresponding author upon reasonable request.
